# T-BET promotes inflammatory specialization without driving terminal maturation of iPSC-derived NK cells

**DOI:** 10.3389/fphar.2026.1952429

**Published:** 2026-10-27

**Authors:** Claire Marsal, Muhammad Kashif, Sophia Borate, Charlotte Hållstrand, Linda Haazen, Mari Gilljam, Yagmur Dölalan, Nutsa Burduli, Stephan Meinke, Anders Norman, Alamdar Hussain, Fredrik Lanner, Ewa Sitnicka, Evren Alici

**Affiliations:** 1 Center for Hematology and Regenerative Medicine, Department of Medicine Huddinge, Huddinge, Sweden; 2 Department of Clinical Sciences, Intervention and Technology, Karolinska Institutet, Stockholm, Sweden; 3 Gynecology and Reproductive Medicine, Karolinska Universitetssjukhuset, Stockholm, Sweden; 4 Lund Stem Cell Center and Division of Molecular Hematology, Department of Laboratory Medicine, Lund University, Lund, Sweden; 5 Hematology Center, Karolinska Universitetssjukhuset, Huddinge, Sweden

**Keywords:** immunotherapy, induced pluripotent stem cells (iPSCs), inflammatory programming, iPSC differentiation, natural killer cells (NK cells), NK cell maturation, off-the-shelf NK cells, T-BET (TBX21)

## Abstract

Induced pluripotent stem cells (iPSCs) provide a scalable platform for off-the-shelf immune cell therapies; however, the mechanisms controlling maturation and functional specialization of iPSC-derived natural killer (iNK) cells are not fully understood. In this study, we combined single-cell transcriptomics, phenotypic profiling, and functional assays to characterize iNK cells and to determine whether temporally controlled expression of the transcription factor T-BET (TBX21) can promote iNK terminal specification and effector functions. Single-cell RNA sequencing and phenotypic profiling confirmed that baseline iNK cells form a transcriptionally distinct NK population and retain a developmental transcriptional signature. These iNK cells exhibited incomplete terminal maturation compared with peripheral blood NK cells, expressing much of the cytotoxic machinery but displaying limited acquisition of maturation-associated receptors. Despite induction of selected terminal specification markers, T-BET did not shift iNK cells toward a peripheral NK-like transcriptional identity. Instead, T-BET selectively triggered an inflammatory effector program characterized by elevated *CCL3*, *CCL5* and *GZMA* expression and increased IFNγ production across multiple tumor targets, without enhancement of terminal cytotoxic gene programs. These findings suggest a functional dissociation between inflammatory and cytotoxic programs in iPSC-derived NK cells. This work highlights transcriptional engineering as a strategy to modulate functional state in stem cell-derived NK cells.

## Graphical Abstract

**Figure:**
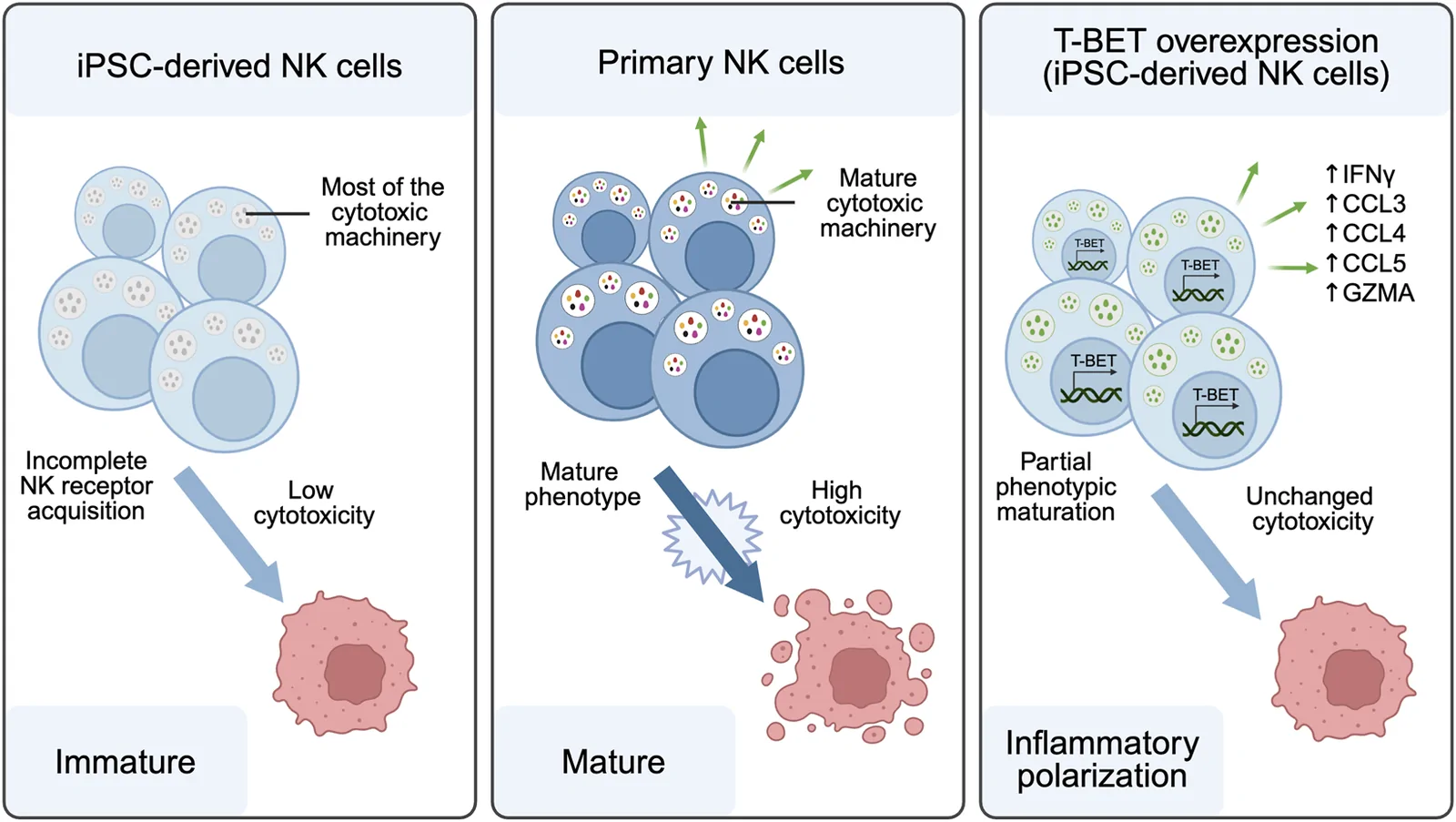

## 1 Introduction

Natural killer (NK) cells are key effectors of the innate immune system and attractive candidates for off-the-shelf cell-based immunotherapy due to their ability to recognize and eliminate malignant cells without prior sensitization and without requiring human leukocyte antigen (HLA) matching (Vivier and Ugolini, 2011; Rezvani and Rouce, 2015). NK cell maturation is generally viewed as a progressive process in which phenotypic maturation and effector function are acquired in parallel (Gordon et al., 2012). According to the linear model proposed by Freud and Caligiuri, NK cells sequentially acquire receptors associated with terminal differentiation, including CD94, killer cell immunoglobulin-like receptors (KIRs), CD16, and CD57, alongside increased cytotoxic and cytokine-producing capacity (Yu et al., 2013). Consistent with this model, Beziat et al. showed that progressive KIR acquisition correlates with terminal maturation and enhanced functional competence (Béziat et al., 2010).

T-BET (TBX21), a T-box transcription factor, is a central regulator of NK cell differentiation and function. Its expression increases along the NK maturation trajectory and is associated with acquisition of effector functions, including interferon gamma (IFNγ) production and expression of cytotoxic molecules such as perforin (PRF1) and granzyme B (GZMB) (Townsend et al., 2004). Beyond its role in transcriptional activation, T-BET has also been shown to regulate *IFNG* through epigenetic and chromatin-level mechanisms (Tong et al., 2005; Sekimata et al., 2009). In addition, T-BET cooperates with transcriptional partners such as eomesodermin (EOMES) (Gordon et al., 2012) and zinc finger E-box-binding homeobox 2 (ZEB2) (van Helden et al., 2015) to regulate terminal NK cell maturation programs.

Expanded peripheral blood-derived NK (PB-NK) cells currently represent the main source of NK cells for clinical applications; however, their use remains limited by donor dependency, batch variability, and scalability constraints (Shimasaki et al., 2020). Induced pluripotent stem cell (iPSC)-derived NK (iNK) cells offer a promising alternative and could provide an unlimited and standardized source for off-the-shelf therapies (Knorr et al., 2013). Nevertheless, current differentiation protocols remain suboptimal. Indeed, iNK cells frequently display an immature phenotype characterized by low expression of terminal maturation markers such as KIRs, CD16, and CD57, together with reduced cytotoxic function compared to PB-NK cells (Huyghe et al., 2024). Transcriptomic analyses further suggest that iNK cells resemble fetal rather than fully mature NK cells, indicating that physiological maturation pathways are not completely recapitulated (Zhang et al., 2024).

Importantly, differentiation strategy influences iNK developmental state. Comparative analyses of feeder-free and OP9 stromal feeder cells expressing the Notch ligand DLL4 (OP9-DLL4)-based protocols demonstrated that both approaches generate incompletely mature NK cells with limited KIR acquisition. Further comparison between feeder-based and feeder-free approaches revealed that feeder-free systems generally yield less mature phenotypic and functional profiles (Huyghe et al., 2024). Despite these limitations, feeder-free protocols provide more defined manufacturing conditions and reduce the risk of feeder-cell contamination, making them attractive for clinical translation (Lupo et al., 2021). Together, these observations highlight the need for strategies that improve terminal specification of feeder-free generated iNK cells *in vitro*.

Despite the established role of T-BET in NK cell maturation, it remains unclear whether supraphysiological T-BET expression can drive terminal maturation of iPSC-derived NK cells. Here, we investigated whether inducible T-BET expression during feeder-free iNK differentiation could promote terminal maturation and functional specialization. Specifically, we sought to determine whether T-BET modulation could partially overcome the maturation constraints of a feeder-free differentiation system.

## 2 Methods

### 2.1 Cell culture

#### 2.1.1 iPSC culture

iPSCs were provided by the iPSC Core Facility at Karolinska Institutet. The three iPSC lines used in this study were generated from fibroblasts of healthy donors using Sendai virus-mediated reprogramming (iPSC7 and iPSC9) or mRNA-mediated reprogramming (iPSC14). Additional characterization of the iPSC lines, including KIR and HLA typing, is provided in Supplementary Figure S9 iPSCs were maintained on iMatrix-coated plates (0.25 μg/cm^2^; Takara, Cat#T304) in mTeSR Plus medium (STEMCELL Technologies, Cat#100–0,276) under standard culture conditions (21% O_2_, 5% CO_2_) and passaged every 3–4 days. For passaging, cells were washed with PBS without Ca^2+^ and Mg^2+^ (Gibco, Cat#14190–094) and dissociated using TrypLE Select (Gibco, Cat#12563–011) for 10 min at 37 °C. Cells were collected as single-cell suspensions, diluted 1:1 with culture medium, and centrifuged at 300 g for 3 min. Following resuspension in fresh medium, viable cells were quantified using a NucleoCounter NC-200 (ChemoMetec) with Via1 cassettes (ChemoMetec, Cat#941–0,012) and seeded at 1–2 × 10^4^ cells/cm^2^ on iMatrix-coated plates in mTeSR Plus supplemented with 5 µM Y-27632 (Tocris Bioscience, Cat#1254). Medium was replaced after 24 h and changed daily thereafter.

#### 2.1.2 PBMC isolation and pNK culture

Peripheral blood mononuclear cells (PBMCs) were isolated from buffy coats of anonymized healthy donors by Ficoll density-gradient centrifugation using Lymphoprep (STEMCELL Technologies, Cat# 07811). All procedures were approved by the Swedish Ethical Review Authority (permit no. 2025–01953–01). Primary NK cells (pNK) were isolated by negative selection (Miltenyi Biotec, Cat#130–092–657), counted using a hemocytometer following Trypan Blue exclusion, and seeded at 1 × 10^6^ cells/mL in CellGenix GMP SCGM medium (Sartorius, Cat# 20802–0,500) supplemented with 10% heated-inactivated human serum (Access Cell Culture, Cat#535-HI GI) and daily addition of 1,000 IU/mL Proleukin (Clinigen), and 10 ng/mL interleukin-15 (IL-15) (Gibco, Cat#200–15). Cells were cultured for 7 days prior to downstream assays.

#### 2.1.3 Cell lines

K562, Raji, and Daudi cell lines were obtained from ATCC. K562 (ATCC,CCL-243) and Raji (ATCC CCL-86) cells were maintained in RPMI 1640 (Invitrogen, Cat#72400021) supplemented with 10% heat-inactivated fetal bovine serum (FBS) (Gibco, Cat#A5256801) and seeded at 3 × 10^5^ cells/mL. Daudi cells (ATCC, CCL-213) were cultured in RPMI 1640 supplemented with 10% non-heat-inactivated FBS (Gibco, Cat#10270).

#### 2.1.4 iPSC nucleofection and cell selection

iPSCs were cultured as described above and nucleofected at 70%–80% confluency using the P3 Primary Cell 4D-Nucleofector X Kit S (Lonza, Cat# V4XP-3032). Briefly, 1 × 10^6^ cells were resuspended in 20 µL P3 buffer and mixed with CAG_HyperPBase, CAG_rtTA_T2A_EBFP2, and either TRE_mCherry_EFS_PuroR (CTRL) or TRE_T-BET_T2A_mCherry_EFS_PuroR (T-BET/OE) (5 µg for each plasmid) (see plasmids details in Supplementary Figure S1C and S1D). Cells were electroporated using program CB-150 on a 4D-Nucleofector system (Lonza, Core Unit Cat#AAF-1003B, Nucleofector X-Unit Cat#AAF-1003X) and plated in mTeSR Plus on iMatrix (0.25 μg/cm^2^) with 5 µM Y-27632. Medium was changed for mTeSR Plus without Y-27632 after 48 h and daily after that. Cells were then passaged as previously described for expansion. On day 17 post-nucleofection, cells were dissociated and sorted by fluorescence-activated cell sorting (FACS; BD FACS Aria Fusion) based on EBFP2 fluorescence. Prior sorting, cells were resuspended in FACS buffer (PBS, 2% FBS) with 1 mM EDTA at 2 × 10^6^ cells/mL, filtered (40 µm), and maintained on ice. Sorted cells were collected in mTeSR Plus supplemented with 5 µM Y-27632 and Antibiotic-Antimycotic (Gibco, Cat# 15240–062), centrifuged (400xg, 3min), and replated at 1–2x10^4^ cells/cm^2^. Medium was changed for mTeSR Plus after 48 h and daily after that. Cells were then passaged as previously described for expansion. At day 10 post sorting, cells were treated with 4 μg/mL of puromycin (Sigma, Cat# P8833-10 mg) in mTeSR Plus over 48 h with daily media change. Following sorting and puromycin selection, CTRL and T-BET/OE cultures were maintained as bulk-selected populations; no single-cell cloning was performed. On day 7 post puromycin selection, to validate activation of inducible system, iPSCs were treated with 1 μg/mL doxycycline for 24 h, followed by staining with Far Red Live Dead (Invitrogen, CAT#3143076), fixation with paraformaldehyde, and flow cytometry analysis of EBFP2 and mCherry expression or intracellular staining for T-BET (BD Biosciences, Cat#561316). Importantly, iPSCs used for validation of inducible system were discarded after characterization and cells used for differentiation experiments were not pre-exposed to doxycycline prior to differentiation. In addition, prior differentiation, the pluripotency and trilineage differentiation potential of the engineered lines were validated (Supplementary Figure S8).

### 2.2 NK cell differentiation from iPSC

The iPSC-to-NK cell differentiation protocol was adapted from a previously described method (Zhu and Kaufman, 2019). For Figure 1, iNK differentiations were performed using three independent iPSC lines (iPSC7, iPSC9, and iPSC14; additional characterization is provided in Supplementary Figure S8D). For Figures 2–5, data were generated using the genetically modified iPSC14 line carrying the inducible expression system. iPSCs were differentiated into NK cells using a spin embryoid body (EB)-based protocol. Briefly, 8 × 10^3^ iPSCs were seeded per well in low-attachment U-bottom 96-well plates (Corning, Cat # CLS7007-24 EA) in STEMdiff APEL2 medium (STEMCELL Technologies, Cat# 1000049837) supplemented with 5 µM Y-27632 (day 0 only), 40 ng/mL stem cell factor (SCF) (Gibco, Cat# 300–07), 20 ng/mL vascular endothelial growth factor (VEGF) (Gibco, Cat# 100-20B), and 20 ng/mL bone morphogenetic protein 4 (BMP-4) (Gibco, Cat# 120–05). On day 1, fresh medium containing cytokines was added, and half-medium changes were performed every other day until day 12. On day 12, EBs were transferred to uncoated 6-well plates and cultured for 5 weeks in NK differentiation medium containing interleukin-3 (IL-3) (5 ng/mL; first week only; Gibco, Cat# 200–03), IL-15 (10 ng/mL; Gibco, Cat# 200–15), interleukin-7 (IL-7) (20 ng/mL; Gibco, Cat# 200–07), SCF (20 ng/mL), and FMS-like tyrosine kinase 3 ligand (FLT3-L) (10 ng/mL; Gibco, Cat# 300–19), with weekly full medium changes. NK basal medium consisted of DMEM/GlutaMAX (Gibco, Cat# 10569010), Ham’s F12 (Gibco, Cat# 31765035), and 15% heat-inactivated human serum, supplemented with L-glutamine (2 mM, Sigma-Aldrich Cat# G7513-100 mL), 2-mercaptoethanol (1µM, Sigma-Aldrich Cat#M3148-25 mL), sodium selenite (5 ng/mL, Sigma-Aldrich Cat# S9133-1 MG), ethanolamine (50 µM), and ascorbic acid (20 μg/mL, Sigma-Aldrich CAT A4544-25G). After differentiation, suspension cells were collected, centrifuged (500xg, 5 min), and cultured for 7 days in SCGM (CellGenix, Cat# 20802–0,500) supplemented with 10% heated-inactivated human serum (Access Cell Culture, Cat#535-HI GI), 1,000 IU/mL Proleukin (Clinigen), and 10 ng/mL IL-15 at 1 × 10^6^ cells/mL. For induction of the inducible system, doxycycline hyclate (1 μg/mL) was added every other day during differentiation.

**FIGURE 1:**
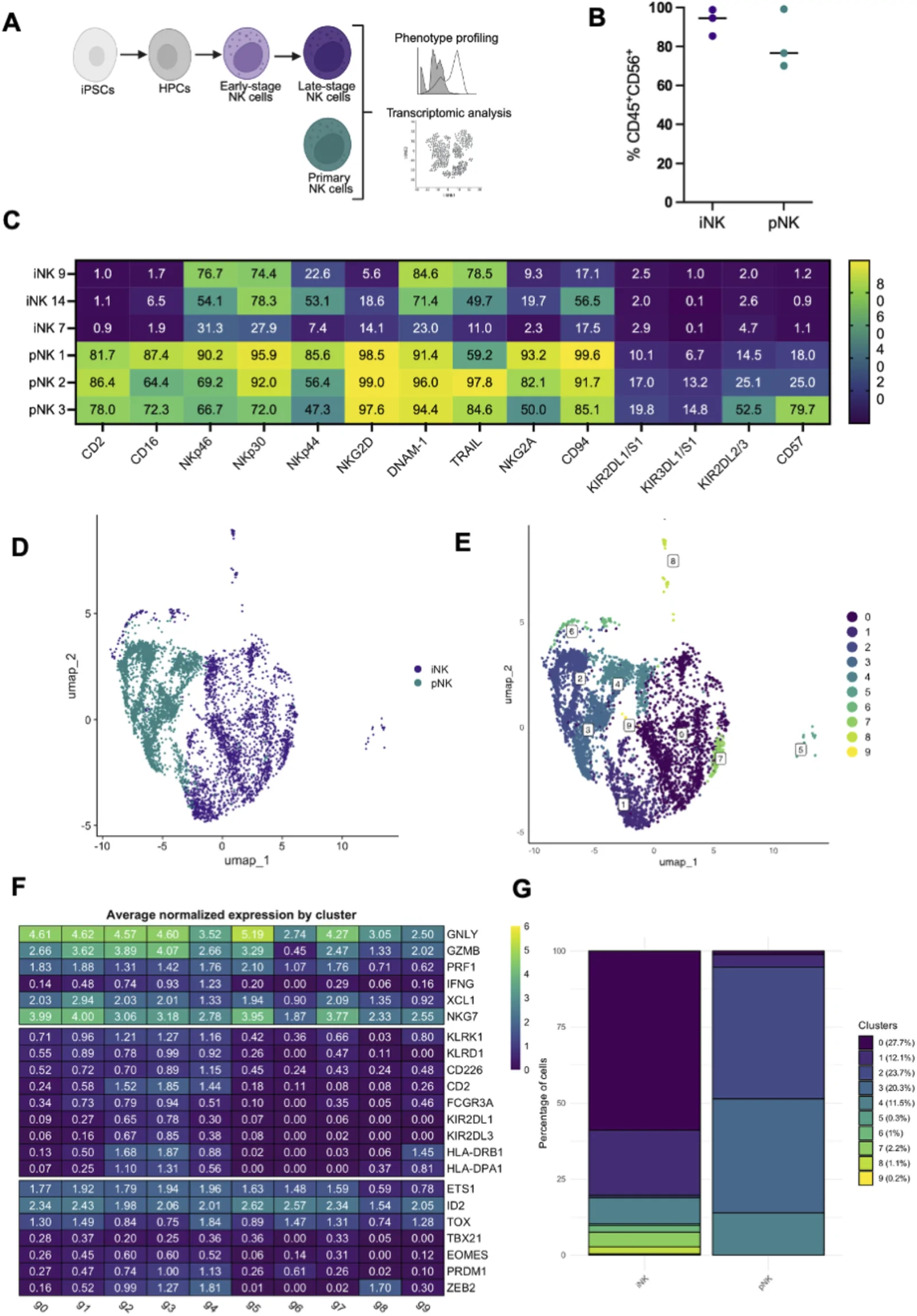
iPSC-derived NK cells exhibit a distinct transcriptional and phenotypic profile compared to primary NK cells. **(A)** Schematic overview of the differentiation workflow from iPSCs to iNK cells. Phenotypic profiling and transcriptomic analyses were performed using pNK cells as reference. **(B)** Frequency of CD45^+^CD56^+^ NK cells in iNK cells (N = 3 iPSC lines differentiated) and pNK cells (N = 3 healthy donors). **(C)** Heatmap showing the percentage of CD45^+^CD56^+^ NK cells expressing indicated surface markers in iNK (N = 3 iPSC line differentiated) and pNK (N = 3 healthy donors) populations. **(D)** UMAP projection of single-cell RNA sequencing data showing the distribution of iNK (N = 1 iPSC line differentiated) and pNK (N = 1 healthy donor) cells. **(E)** Unsupervised clustering of single-cell transcriptomes visualized by UMAP, with clusters annotated numerically. **(F)** Heatmap showing average normalized expression of selected NK cytotoxicity-, receptor- and transcription factor -associated genes across identified clusters. Selected receptor aliases are: *KLRK1* (NKG2D), *KLRD1* (CD94), *FCGR3A* (CD16), and *CD226* (DNAM-1) **(G)** Relative distribution of iNK and pNK cells across transcriptionally defined clusters identified by unsupervised clustering analysis. Data in **(B)** are shown as individual values with mean. Heatmaps in **(C,F)** represent mean of frequency or expression values. **(B)** No statistical significance was identified using the Mann-Whitney test (two-tailed, unpaired) (p > 0.05).

**FIGURE 2:**
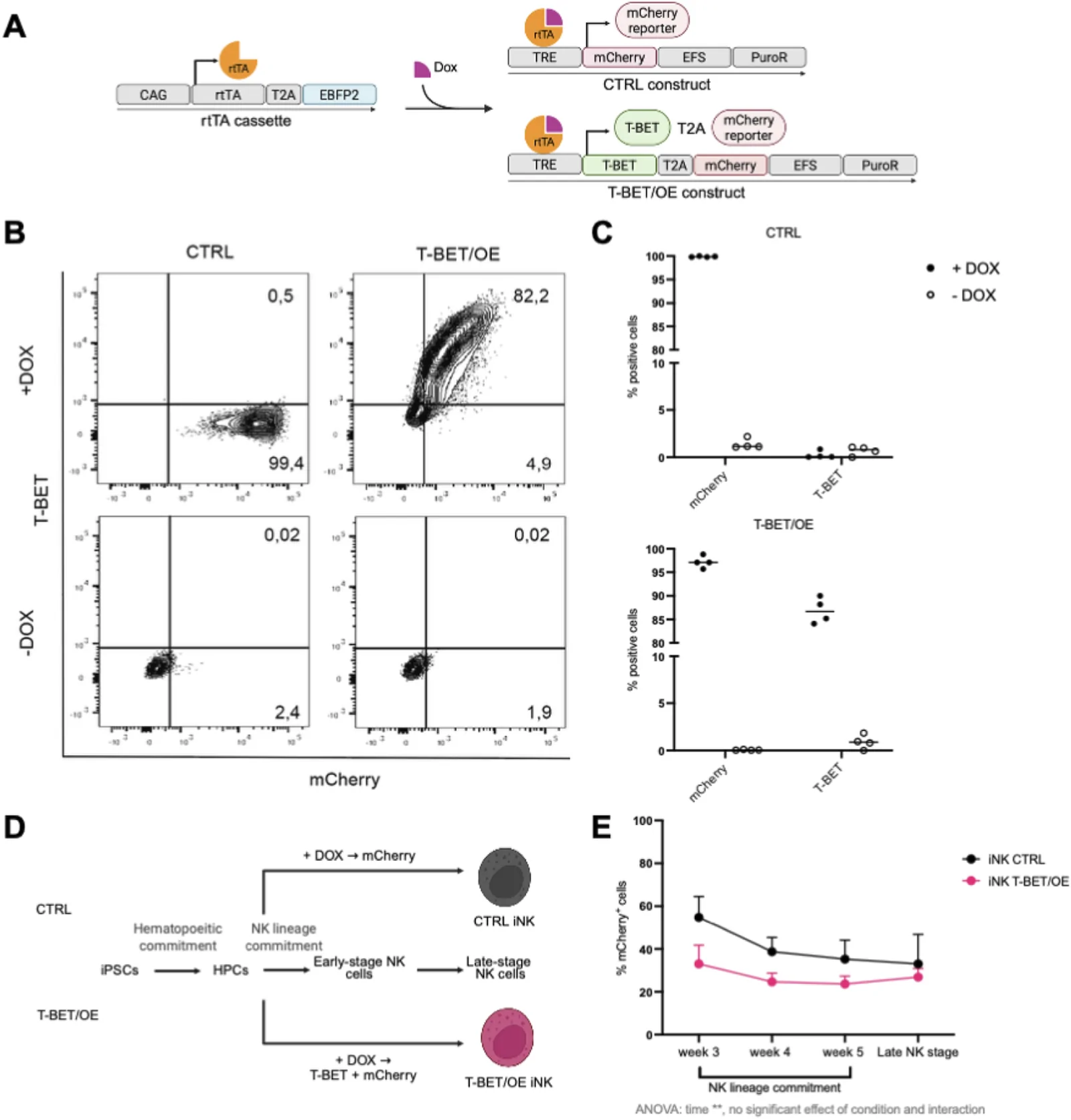
Establishment of an inducible T-BET overexpression system in iPSC-derived NK cells **(A)** Schematic representation of the doxycycline-inducible system. The rtTA cassette is driven by a constitutive promoter, enabling doxycycline-dependent activation of a TRE-controlled construct expressing either mCherry alone (CTRL) or T-BET and mCherry (T-BET/OE). **(B)** Representative flow cytometry plots showing T-BET expression and mCherry reporter activity in CTRL and T-BET/OE iPSCs cultured in the presence (+DOX) or absence (-DOX) of doxycycline for 24 h. **(C)** Quantification of mCherry and T-BET positive cells in CTRL and T-BET/OE iPSCs with (+DOX) or without (-DOX) doxycycline exposure for 24 h (N = 4 independent experiments). **(D)** Schematic overview of the differentiation timeline and experimental setup for induction of T-BET and/or mCherry expression during NK cell differentiation from iPSCs. **(E)** Percentage of mCherry-positive cells at different stages of NK cell differentiation in CTRL (N = 3 independent differentiations of one engineered iPSC line) and T-BET/OE (N = 3 independent differentiations of one engineered iPSC line). Statistical analysis was performed using a two-way repeated-measures ANOVA is reported below the graph. No statistically significant pairwise comparisons were identified by Šídák’s multiple comparisons test (p > 0.05). Data in **(C,E)** are shown as individual values with mean ± SD.

**FIGURE 3:**
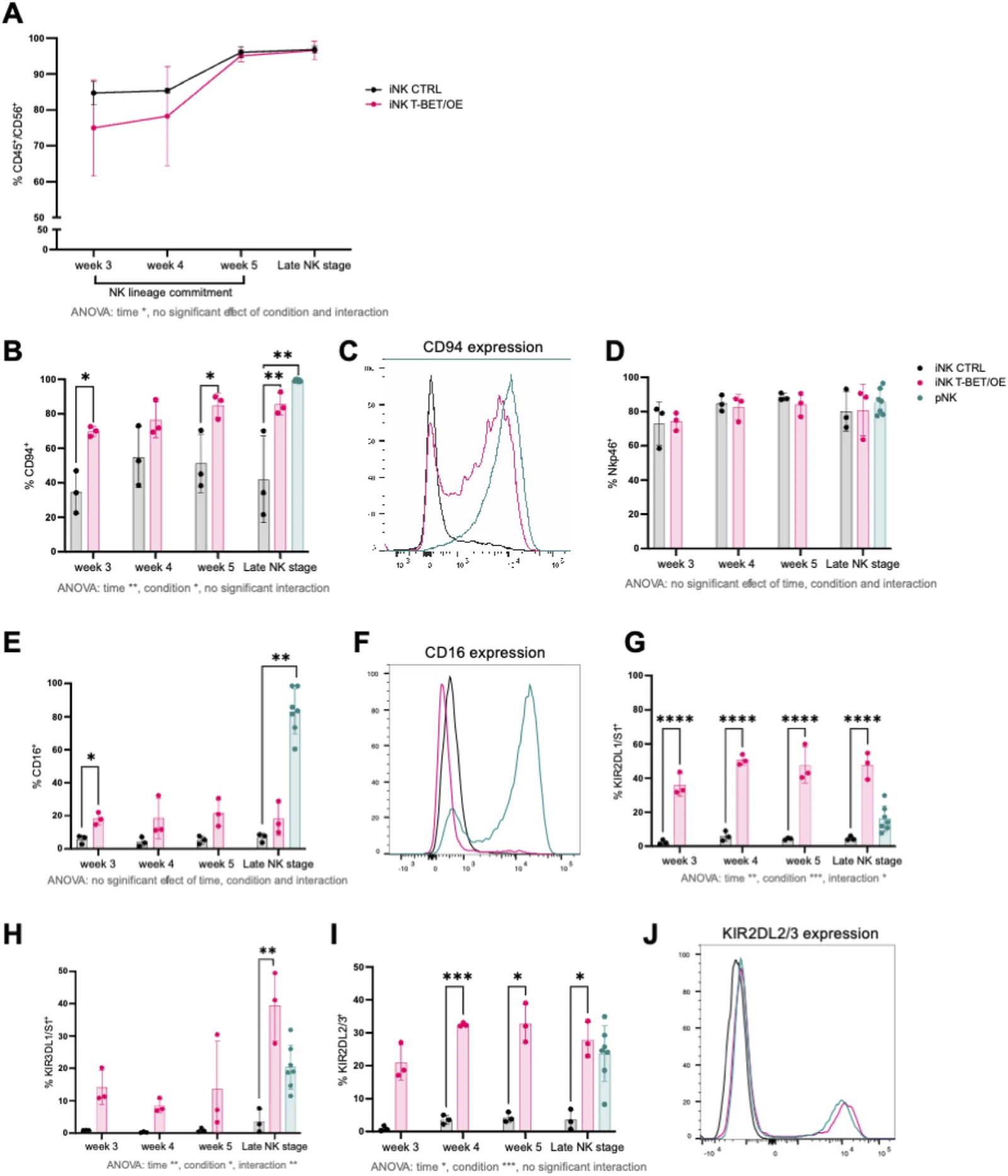
T-BET modulates phenotypic maturation without inducing terminal NK maturation. **(A)** Frequency of CD45^+^CD56^+^ cells across differentiation stages (weeks 3–5 of NK lineage commitment and late NK stage) in iNK CTRL and iNK T-BET/OE. **(B)** Percentage of CD94^+^ cells across differentiation stages in iNK CTRL, iNK T-BET/OE, and pNK cells. **(C)** Representative histogram of CD94 expression at the late NK stage. **(D,E)** Percentage of NKp46^+^ **(D)** and CD16^+^ **(E)** cells across differentiation stages. **(F)** Representative histogram of CD16 expression at the late NK stage. **(G–I)** Percentage of KIR2DL1/S1^+^ **(G)**, KIR3DL1/S1^+^ **(H)**, and KIR2DL2/3^+^ **(I)** cells across differentiation stages. **(J)** Representative histogram of KIR2DL2/3 expression at the late NK stage. Data are presented as mean ± SD. iNK CTRL (N = 3 independent differentiations of one engineered iPSC line), iNK T-BET/OE (N = 3 independent differentiations of one engineered iPSC line), and pNK (N = 7 healthy donors). Overall statistical significance determined by the two-way repeated-measures ANOVA is reported below each graph. Statistically significant pairwise comparisons identified by Šídák’s or Dunn’s multiple comparisons tests are indicated on the graphs using connecting lines and asterisks (*p* < 0.05 (*), p < 0.01 (**), *p* < 0.001 (***), p < 0.0001 (****).

**FIGURE 4:**
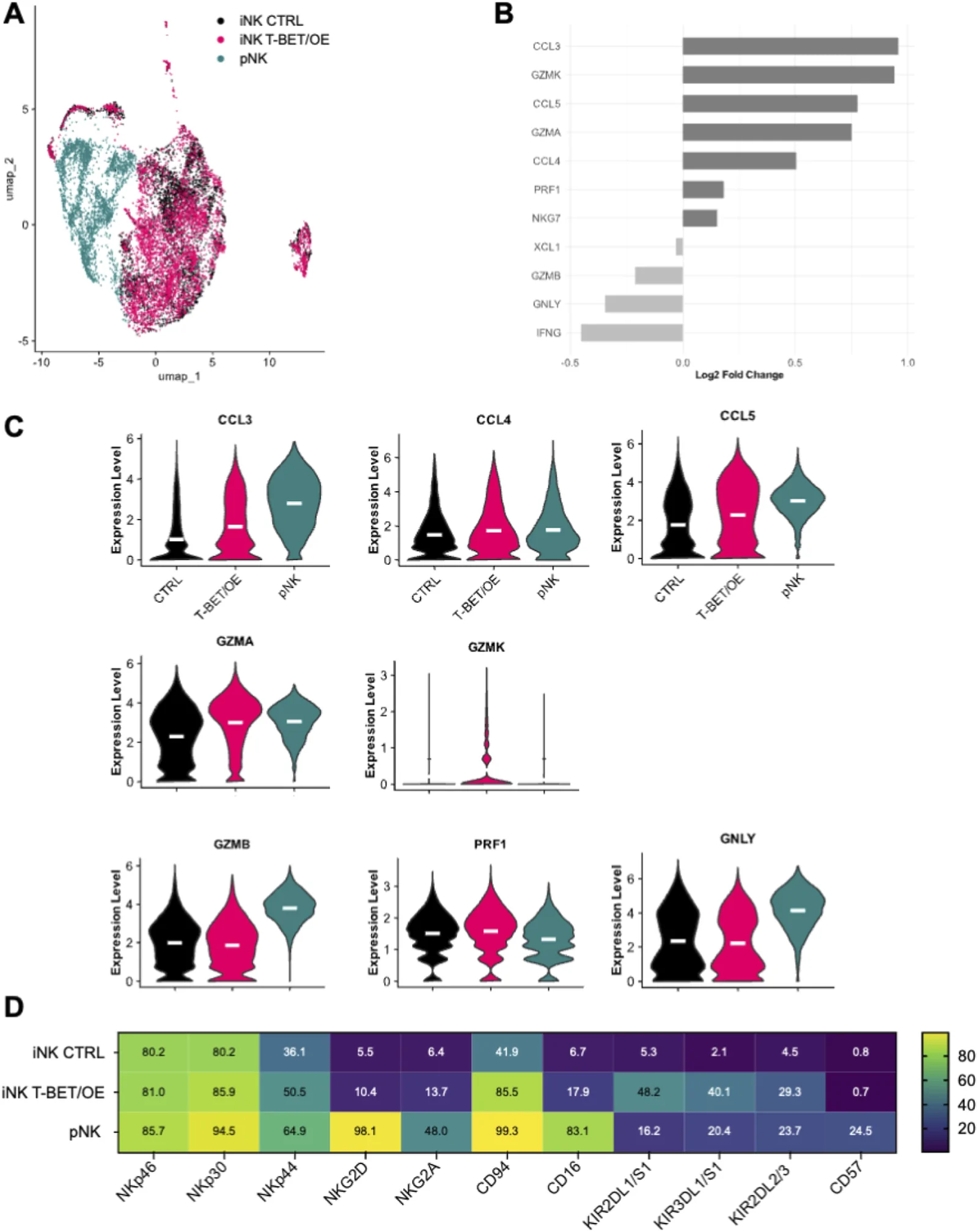
T-BET induces a pro-inflammatory transcriptional program without promoting terminal cytotoxic differentiation. **(A)** UMAP visualization of single-cell RNA-seq profiles of iNK CTRL, iNK T-BET/OE, and pNK cells. **(B)** Differential gene expression analysis (log_2_ fold change) between iNK T-BET/OE and iNK CTRL cells highlighting inflammatory and cytotoxic gene signatures. **(C)** Violin plots showing expression of selected genes associated with inflammatory function (*CCL3, CCL4, CCL5, GZMA, GZMK*) and cytotoxic machinery (*GZMB, PRF1, GNLY, NKG7*). **(D)** Heatmap of surface receptor expression across iNK CTRL, iNK T-BET/OE, and pNK cells.For scRNA-seq analyses **(A–C)**, data are derived from one representative experiment (N = 1 differentiation for CTRL and T-BET/OE and 1 healthy donor for pNK). For phenotypic analysis **(D)**, data represent mean values from iNK CTRL (N = 3 independent differentiations of one engineered iPSC line), iNK T-BET/OE (N = 3 independent differentiations of one engineered iPSC line), and pNK (N = 7 healthy donors).

**FIGURE 5:**
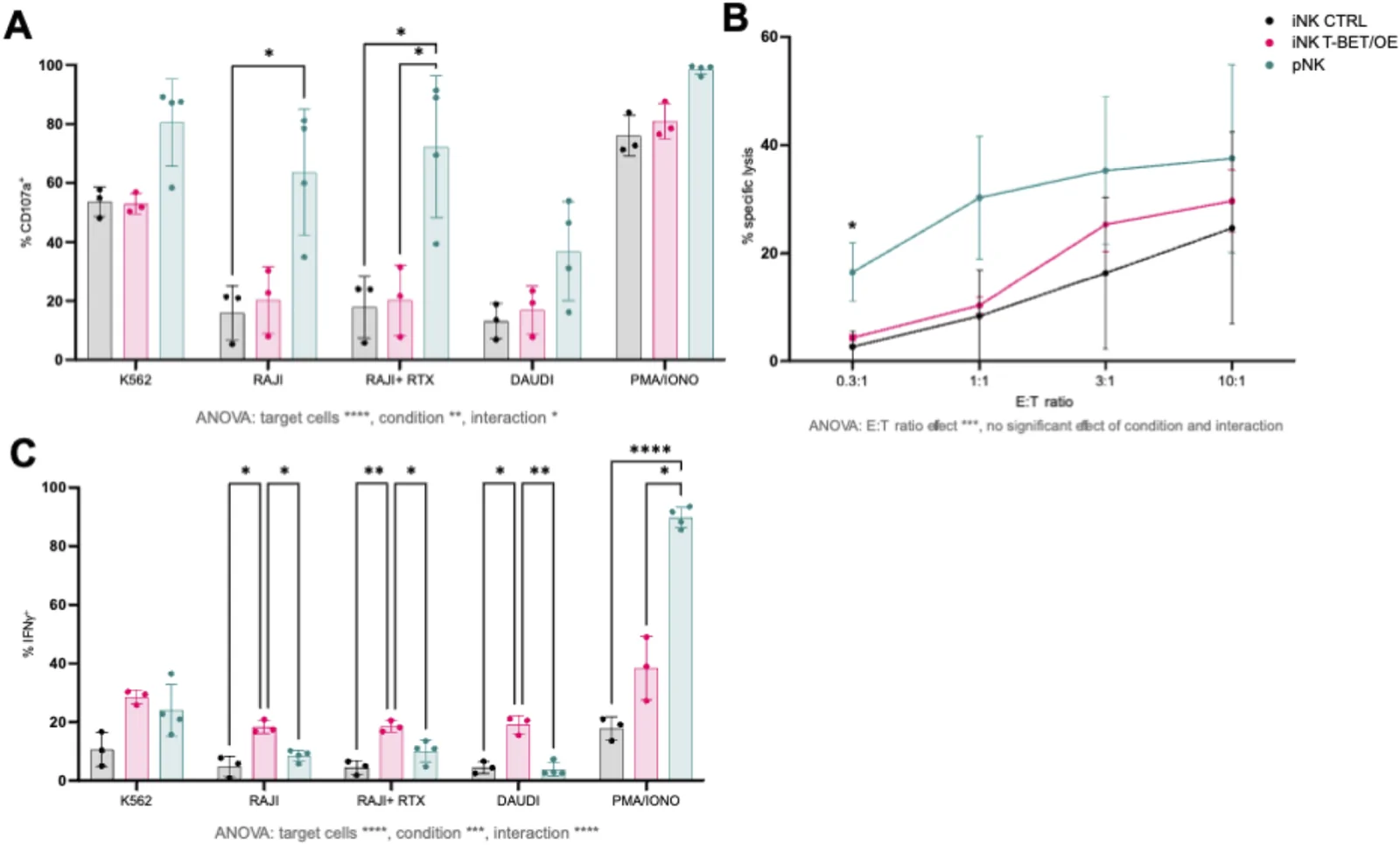
T-BET promotes IFNγ production without enhancing cytotoxic function in iNK cells **(A)** Degranulation activity measured by CD107a expression in iNK CTRL, iNK T-BET/OE, and primary NK (pNK) cells following co-culture with target cells (K562, RAJI, RAJI + RTX, DAUDI) or stimulation with PMA/ionomycin. **(B)** Cytotoxic activity assessed by specific lysis of target cells labelled with chromium-51 at indicated effector-to-target (E:T) ratios. Šídák’s multiple comparisons test identified a statistically significant difference only between iNK CTRL and pNK at an E:T ratio of 0.3:1 (*P* < 0.05); no other pairwise comparisons reached statistical significance. **(C)** IFNγ production in iNK CTRL, iNK T-BET/OE, and pNK cells following stimulation with target cells or PMA/ionomycin. Data are shown as mean ± SD. iNK CTRL, N = 3 independent differentiations of one engineered iPSC line; iNK T-BET/OE, N = 3 independent differentiations of one engineered iPSC line; pNK, N = 4 healthy donors. Overall statistical significance determined by the two-way repeated-measures ANOVA is reported below the graph. Statistically significant pairwise comparisons identified by Šídák’s multiple comparisons test are indicated by asterisks as follows: *p* < 0.05 (*), p < 0.01 (**), *p* < 0.001 (***), p < 0.0001 (****).

### 2.3 Flow cytometry staining and analysis

Staining was performed according to established guidelines for flow cytometry (Cossarizza et al., 2021). For extracellular staining, cells were washed once in PBS and incubated with Live/Dead Aqua dye (Invitrogen, Cat# 3240491) for 15 min at 4 °C in the dark. Cells were then washed and stained with surface antibodies in FACS buffer (PBS supplemented with 2% FBS) for 30 min at 4 °C in the dark. Following staining, cells were washed and fixed with 1% paraformaldehyde (Histolab, Cat# 02176) for 10 min, washed again, and resuspended in FACS buffer. For intranuclear staining, cells were fixed and permeabilized using 1x Fixation/Permeabilization solution (Invitrogen, Cat# 00–5,123–43 and 00–5,223–56) for 30 min at room temperature in the dark. Cells were then washed with 1x Perm/Wash buffer (Invitrogen, Cat# 00–8,333–56) and stained with intranuclear antibodies for 30 min at room temperature in the dark. Following staining, cells were washed and resuspended in FACS buffer. All antibodies used in the study are shown in Supplementary Tables S1. Data acquisition was performed on a BD Symphony A5 SORP (BD Biosciences), and analysis was carried out using FlowJo software (v10, Tree Star). Representative gating strategies are shown in Supplementary Figures S5 and S6.

### 2.4 Functional assays

Functional assays were performed on iNK cells at the end of differentiation and on pNK cells after 1 week of culture post isolation under identical culture conditions.

#### 2.4.1 Degranulation assay

Effector cells (1 × 10^5^ cells/well) were co-cultured with target cells (1 × 10^5^ cells/well) at a 1:1 ratio in 96-well U-bottom plates (Corning, Cat# 3799). Where indicated, rituximab (Absolute antibodies, Cat# Ab00126-10) was added at 2.5 μg/mL. Control conditions included effector cells alone or with phorbol 12-myristate 13-acetate (PMA) (50 ng/mL, Sigma-Aldrich Cat#P1585) and ionomycin (IONO) (500 ng/mL, Sigma-Aldrich Cat # I9657). All conditions were incubated in the presence of anti-CD107a antibody (BioLegend Cat# 328618) at 37 °C for 4 h. After 1 h, monensin (GolgiStop; BD Biosciences, Cat# 554724) was added, and cells were incubated for an additional 3 h. Following incubation, cells were stained for surface markers as described above and for intracellular IFNγ (BioLegend, Cat# 506510) using the Cytofix/Cytoperm kit (BD Biosciences, Cat# 554714) according to the manufacturer’s instructions. Experiments were performed in technical duplicates for iNK T-BET/OE and pNK cells, and in singlets for iNK CTRL cells.

#### 2.4.2 Chromium release assay

NK cell-mediated cytotoxicity was assessed using a chromium-51 (51Cr)-release assay against K562 target cells. Briefly, target cells were labeled with 51Cr (Revity, Cat# NEZ030005MC) for 1 h at 37 °C and washed three times with PBS. Labeled target cells (3 × 10^3^ cells/well) were co-cultured with NK cells in 96-well V-bottom plates at effector-to-target (E:T) ratios of 10:1, 3:1, 1:1, and 0.3:1 for 4 h at 37 °C. Spontaneous release was determined from target cells cultured alone, and maximum release from target cells lysed with Triton X-100 (0.5%) (Bio-Rad Laboratories AB, Cat#1610407). After incubation, 20 µL of supernatant was transferred to LumaPlate-96 plates (Revity, Cat# 6006633) and radioactivity was measured using a MicroBeta2 counter (PerkinElmer). Experiments were performed in duplicates. Percentage of specific lysis per sample type = [(experimental - spontaneous release)/(maximum load - spontaneous release) x 100].

### 2.5 Single cell RNAseq

Regarding cell selection, iPSC-derived NK cells from CTRL and T-BET/OE conditions were dissociated into single-cell suspensions, resuspended in FACS buffer with 1 mM EDTA at 2 × 10^6^ cells/mL, filtered through a 40 µm strainer, and maintained on ice. Cells were sorted by fluorescence-activated cell sorting (FACS; BD FACS Aria Fusion) based on mCherry expression using wild-type controls for gating. Sorted cells were collected in NK medium (CellGenix GMP SCGM medium supplemented with 10% heated-inactivated human serum), centrifuged (400×g, 3 min), and resuspended in PBS at 1,500 cells/mL. Single-cell RNA-seq (scRNAseq) libraries were generated using the Chromium X platform and GEM-X Universal 3′Gene Expression v4 kit (10x Genomics, PN-1000691) according to the manufacturer’s instructions. Briefly, single cells were partitioned into Gel Beads-in-Emulsions (GEMs) for barcoding and reverse transcription, followed by cDNA amplification and library preparation. Libraries were sequenced on an Illumina platform. Single cell and library preparation were performed by the Single Cell Core Facility (SICOF) and sequencing was performed by the Bioinformatics and Expression Analysis (BEA) core facility at Karolinska Institutet.

Regarding analysis, raw sequencing reads were demultiplexed and aligned to the human reference genome (gex-GRCh38-2024-A) using Cell Ranger (version 9.0.0). The resulting single-cell UMI count matrices were imported into R (v4.5.2) and analyzed using the Seurat package (v5.4.0). Quality control filtering was applied to remove low-quality cells (Supplementary Figure S7). Cells with fewer than 500 or more than 6,000 detected genes (nFeature_RNA), more than 25,000 UMI counts (nCount_RNA), or greater than 10% mitochondrial transcript content were excluded from downstream analyses. After filtering, data were normalized using the SCT transform variance-stabilizing workflow. Principal component analysis (PCA) was performed using the top variable genes, followed by construction of a shared nearest neighbor (SNN) graph. Cell clustering was carried out using the Louvain algorithm with a resolution parameter of 0.20 and UMAP was subsequently performed using the first 25 principal components. Differential gene expression analysis was performed using the FindMarkers function and average expression of selected gene signatures was calculated using Seurat’s AverageExpression function that were visualized as heatmaps to compare functional states across cell populations.

### 2.6 Statistical analysis

Statistical analyses were performed using GraphPad Prism (version 10, GraphPad Software). Data are presented as mean ± standard deviation (SD) unless otherwise indicated. For Figure 1B, statistical comparisons between two groups were performed using a two-tailed Mann-Whitney test. A non-parametric test was selected due to the small sample size and because normal distribution could not be assumed. For Figures 2–5, experiments involving multiple time points or conditions were analyzed using two-way repeated measures ANOVA followed by Šídák’s multiple comparisons test. Comparisons between more than two groups at a single time point were performed using a non-parametric Kruskal–Wallis test followed by Dunn’s multiple comparisons test. Only statistically significant comparisons are indicated in figures. A p value <0.05 was considered statistically significant.

## 3 Results

### 3.1 iPSC-derived NK cells display an immature phenotype compared to primary NK cells

iPSC lines from healthy donors were differentiated into NK cells using a feeder-free embryoid body-based protocol. Peripheral NK (pNK) cells were isolated from healthy donor buffy coats and cultured for 1 week under conditions matching the late-stage iNK differentiation protocol. Both cell populations were subsequently characterized by flow cytometry and single-cell RNA sequencing (scRNA-seq) (Figure 1A).

The frequency of CD45^+^CD56^+^ NK cells was comparable between iNK and pNK cultures (Figure 1B). However, phenotypic analysis revealed clear differences in NK maturation markers. Compared with pNK cells, iNK cells expressed lower levels of CD16, CD94, KIRs and CD57, together with differences in activating receptors including NKp44, NKG2D and DNAM-1, consistent with an incompletely mature phenotype (Figure 1C).

UMAP analysis revealed that iNK and pNK cells occupied distinct transcriptional spaces (Figure 1D; Supplementary Figure S1A–C), while unsupervised clustering identified 10 transcriptionally distinct populations (Figure 1E). Clusters 0 and 1 consisted almost exclusively of iNK cells, whereas clusters 2 and 3 were predominantly composed of pNK cells. Cluster 4 contained both iNK and pNK cells, while the remaining clusters represented minor cell subsets (Figure 1G). Analysis including additionnal pNK donors is shown in Supplementary Figure S1A–C. The top differentially expressed genes between iNK and pNK cells, as well as the top genes driving clusters separation, are shown in Supplementary Figure S1D–E.

Expression of canonical NK-associated genes revealed substantial heterogeneity across clusters (Figure 1F). Cytotoxic effector genes, including *GNLY, PRF1, GZMB, XCL1* and *NKG7*, were expressed in both iNK- and pNK-enriched clusters. Notably, clusters 0 and 1 expressed *GNLY, PRF1* and *NKG7* at levels comparable to (for *GLNY*), or higher (for *PRF1* and *NKG7*) than those observed in pNK-enriched clusters. In contrast, *GZMB* expression was reduced in cluster 0, whereas *XCL1* was enriched in cluster 1. Cluster 4 displayed lower expression of several cytotoxic effector genes than the major iNK- and pNK-enriched clusters. Expression of *FCGR3A* (CD16), *KLRD1* (CD94), *KLRK1* (NKG2D), *CD2*, multiple *KIR* genes, and *HLA* genes was lowest in cluster 0, whereas cluster 1 displayed intermediate expression of several maturation markers, including CD94 and CD16. Cluster 4 more closely resembled pNK-enriched clusters, although CD16, *KIR* genes and *HLA* class II genes (*HLA-DPA1* and *HLA-DRB1*) remained expressed at lower levels.

A similar gradient was observed for transcription factors regulating NK-cell maturation. Clusters 0 and 1 expressed higher levels of early NK progenitor-associated TF such as *ID2* and *TOX*, while more mature NK-associated TF like *PRDM1* (Blimp-1), *EOMES* and *ZEB2* were reduced compared with pNK-enriched clusters. Cluster 1 exhibited intermediate expression of *PRDM1*, *EOMES* and *ZEB2* relative to clusters 0 and 2–3. Likewise, cluster 4 displayed a mixed profile, combining pNK-like *ID2* and *PRDM1* expression with persistent *TOX* expression and relatively high *ZEB2* levels.

Together, these data indicate that iPSC-derived NK cells acquire expression of major cytotoxic effector molecules while remaining transcriptionally distinct from peripheral NK cells, primarily through incomplete acquisition of maturation-associated NK receptors and transcription factor networks.

### 3.2 Establishment and validation of an inducible T-BET overexpression system in iPSC-derived NK cells

Given the established role of the transcription factor T-BET in NK cell maturation, we sought to determine whether its overexpression during iPSC-derived NK cell differentiation could enhance maturation and functional properties.

To this end, we developed a doxycycline-inducible T-BET overexpression system using a piggyBac-based genomic integration strategy at the iPSC stage. The system consists of two constructs: (I) a rtTA cassette enabling constitutive expression of the reverse tetracycline transactivator (rtTA) and (II) a tetracycline response element (TRE)-driven construct expressing either mCherry alone (CTRL) or T-BET together with mCherry (T-BET overexpression, hereafter referred to as T-BET/OE) via a T2A sequence. In this inducible system, mCherry was used as a reporter of system activation. Indeed, upon doxycycline exposure, rtTA activates the TRE promoter, inducing expression of mCherry in CTRL cells and co-expression of T-BET and mCherry in T-BET/OE cells (Figure 2A). The plasmids used are presented in Supplementary Figure S2A–D.

Following cell selection, the inducible system was validated in iPSCs. Doxycycline treatment resulted in robust induction of mCherry expression in nearly all cells in both conditions, while T-BET expression was detected specifically in the T-BET/OE condition. In the absence of doxycycline, minimal background expression of mCherry or T-BET was observed, indicating tight control of the system (Figures 2B,C; Supplementary Figure S2E).

Although the percentage of T-BET^+^ cells (mean 87%) was slightly lower than that of mCherry^+^ cells (mean 97%) in the T-BET/OE condition, this likely reflects technical limitations associated with intracellular staining of transcription factors (Figure 2C). Given the bicistronic design of the construct, mCherry expression reliably reports T-BET induction (Figure 2A).

To assess inducible system functionality during differentiation, iPSCs carrying the CTRL or T-BET/OE constructs were differentiated into iNK cells, with doxycycline added from the onset of NK lineage commitment (Figure 2D). mCherry expression was maintained throughout differentiation, with 20%–65% of cells expressing the reporter across time points (Figure 2E).

For most downstream phenotypic, functional (except 51Cr-release assay), and transcriptomic analyses, mCherry^+^ cells were selectively analyzed or sorted to ensure assessment of cells with active transgene expression. Overall, these data indicate that our doxycyline-inducible system allow targeted and controlled transcriptional modulation of NK cell differentiation.

### 3.3 T-BET modulates phenotypic maturation without inducing terminal NK maturation

We next assessed the impact of T-BET overexpression on iNK cell differentiation kinetics and phenotypic maturation at different stages of NK lineage commitment and after late NK cell differentiation using flow cytometry (Figure 3). Total suspension cell yields at the end of NK lineage commitment are also shown in Supplementary Figure S3C.

The percentage of CD45^+^CD56^+^ cells increased over time in both conditions, reflecting normal NK lineage commitment. However, no significant differences were observed between iNK CTRL and iNK T-BET/OE cells at any time point, indicating that T-BET overexpression does not affect early NK cell commitment (Figure 3A).

We then examined markers associated with early NK cell maturation. While NKp46 expression remained unaffected across conditions and time points (Figure 3D), T-BET overexpression significantly increased the percentages of CD94^+^ cells over time (Figures 3B,C; Supplementary Figure S3D). At the late NK stage, CD94 expression in iNK T-BET/OE cells (mean 86%) reached levels comparable to primary NK (pNK) cells (mean 99%), whereas iNK CTRL cells displayed significantly lower CD94 expression (mean 42%) (Figure 3B).

We next analyzed markers associated with terminal NK cell maturation. CD16 expression remained low both in iNK CTRL and iNK T-BET/OE cells across all time points, with no significant effect of T-BET overexpression despite a transient increase at week 3 (Figures 3E,F; Supplementary Figure S3D). In contrast, T-BET overexpression strongly induced the expression of multiple KIRs (KIR2DL1/S1, KIR3DL1/S1, KIR2DL2/3) as early as week 3 of NK lineage commitment, whereas these receptors were minimally expressed in iNK CTRL cells (Figures 3G–J; Supplementary Figure S3D). Additional phenotypic markers are shown in Supplementary Figure S3A and SB.

Overall, these results indicate that T-BET overexpression enhances aspects of NK cell maturation, as reflected by increased CD94 and KIRs expression. However, the low acquisition of CD16 suggests that T-BET alone is insufficient to drive full terminal maturation of iNK cells.

### 3.4 T-BET induces a pro-inflammatory transcriptional program without promoting terminal cytotoxic differentiation

We next compared iNK CTRL, iNK T-BET/OE and pNK cells at the late NK cell stage using combined phenotypic and transcriptomic analyses (Figure 4).

UMAP revealed a clear separation between iNK and pNK cells, regardless of T-BET overexpression. Notably, iNK CTRL and iNK T-BET/OE cells largely overlapped, indicating that T-BET overexpression does not induce a global transcriptional shift sufficient to recapitulate the primary NK cell state (Figure 4A).

Differential gene expression analysis identified a selective upregulation of inflammatory and effector-associated genes in iNK T-BET/OE cells. Pro-inflammatory chemokines (*CCL3*, *CCL4*, *CCL5*) as well as granzyme A (*GZMA*) expression were increased, with moderate to strong log_2_ fold changes. A similar increase was observed for *GZMK*, although its expression was detected in only a small fraction of cells and should therefore be interpreted with caution. In contrast, expression of *IFNG* was moderately decreased by T-BET overexpression (log_2_FC < ∼0.5) (Figure 4B). Violin plot visualization confirmed that the increased expression of *CCL3, CCL4, CCL5* and *GZMA* was distributed across a broad fraction of cells rather than being restricted to a small subset. In contrast, *GZMK* expression was detected in only a limited fraction of T-BET/OE cells (Figure 4C).

In contrast, genes associated with terminal cytotoxic function, including *GZMB*, *PRF1* and *GNLY*, showed minimal changes upon T-BET overexpression, with low log_2_ fold changes (log_2_FC < ∼0.5) (Figure 4B). Consistently, violin plots demonstrated largely overlapping expression distributions between iNK CTRL and iNK T-BET/OE cells for these genes (Figure 4C).

When compared to pNK cells, both iNK CTRL and iNK T-BET/OE cells exhibited lower expression of selected cytotoxic genes such as *GZMB* and *GNLY*, consistent with an incompletely mature transcriptional state. However, T-BET overexpression partially shifted iNK cells toward a more inflammatory, pNK-like profile, as reflected by increased expression of *CCL3*, *CCL5* and *GZMA* (Figure 4C).

Phenotypic analysis of CD45^+^CD56^+^ cells further supported these findings. T-BET overexpression increased the expression of CD94 and multiple KIRs, while CD16 and CD57 remained low in both conditions. Expression of other activating and inhibitory receptors was not markedly affected (Figure 4D). Compared to pNK cells, both iNK CTRL and iNK T-BET/OE retained an immature phenotype, although T-BET/OE cells displayed partial maturation features. Additional transcriptomic and phenotypic characterization of iNK CTRL, iNK T-BET/OE and pNK, including an integrated UMAP analysis, with additional pNK donors are shown in Supplementary Figure S4.

Taken together, these data demonstrate that T-BET overexpression promotes a pro-inflammatory transcriptional program and partial phenotypic maturation in iNK cells without inducing full terminal cytotoxic differentiation, supporting a functional dissociation between inflammatory specialization and terminal cytotoxic maturation in iNK cells.

### 3.5 T-BET promotes IFNγ production without enhancing cytotoxic function in iNK cells

We next evaluated NK cell functional responses by assessing degranulation, cytotoxicity and cytokine production following stimulation by different target cells (Figures 5A–C).

Degranulation capacity, measured by CD107a surface expression at a 1:1 effector-to-target ratio, was comparable between iNK CTRL and iNK T-BET/OE cells across all target conditions (Figure 5A), indicating that T-BET overexpression did not significantly improve cytotoxic function under the conditions tested. When compared to pNK cells, iNK CTRL cells exhibited significantly lower degranulation in response to RAJI cells, and both iNK CTRL and iNK T-BET/OE showed reduced responses in the presence of rituximab (RAJI + RTX). This reduced activity in the presence of rituximab-coated targets is consistent with the low frequency of CD16^+^ cells in iNK populations (Figure 4D), as CD16 mediates Fc receptor-dependent recognition required for antibody-dependent cellular cytotoxicity (ADCC).

Then, to directly assess killing capacity, we performed 51Cr-release assays using K562 target cells across increasing E:T ratios (Figure 5B). Cytotoxicity increased with higher E:T ratios in all conditions. Compared to pNK cells, iNK CTRL cells exhibited significantly reduced cytotoxicity at the lowest E:T ratio tested (0.3:1), whereas no significant differences were observed between iNK T-BET/OE and pNK cells or between iNK CTRL and iNK T-BET/OE cells. At higher E:T ratios, iNK T-BET/OE cells showed a numerical increase in cytotoxicity compared with iNK CTRL cells, although this difference did not reach statistical significance. A two-way repeated-measures ANOVA revealed a significant effect of E:T ratio, but no effect of condition or interaction, indicating that T-BET overexpression did not significantly improve cytotoxic function under the conditions tested.

Interestingly, T-BET overexpression increased the frequency of IFNγ-positive iNK cells following stimulation with Raji, rituximab-coated Raji, and Daudi cells. A numerical increase was also observed following K562 stimulation, but this comparison did not reach statistical significance. T-BET/OE cells also showed a higher frequency of IFNγ positivity than pNK cells after Raji, rituximab-coated Raji, and Daudi stimulation (Figure 5C).

Taken together, these results demonstrate that T-BET overexpression does not significantly enhance degranulation or killing capacity under the conditions tested, but selectively amplifies inflammatory cytokine (IFNγ) production. This supports a functional dissociation between IFNγ production and cytotoxic activity in iNK cells.

### 3.6 Conceptual model of T-BET-driven NK cell positioning along maturation and inflammatory axes

In the classical linear model of NK cell development, immature NK cells progressively differentiate into fully mature cytotoxic NK cells, with phenotypic maturation and effector function increasing in parallel. Fully mature NK cells are characterized by high expression of CD94, CD16 and KIRs, as well as by acquisition of CD57, and exhibit strong cytotoxic activity associated with high expression of cytotoxic molecules, including *GZMB* and *GNLY* (Yu et al., 2013; Béziat et al., 2010).

In contrast, iPSC-derived NK cells (iNK CTRL) display an intermediate maturation state. At the transcriptional level, they exhibit incomplete acquisition of the terminal cytotoxic transcriptional program, with lower expression of *GZMB* and *GNLY* despite preserved expression of several other cytotoxic molecules (Figure 4C). Functionally, iNK CTRL cells tend to show lower degranulation and cytotoxicity compared to primary NK cells, although these differences are not consistently significant across all conditions (Figures 5A,B). Phenotypically, iNK CTRL cells are characterized by low expression of maturation-associated markers, including CD94, CD16 and KIRs, and by lack CD57 expression (Figure 4D). The reduced expression of CD16 likely contributes to impaired ADCC, while the functional implications of reduced CD94, KIR and CD57 expression remain less clearly defined.

T-BET overexpression shifts iNK cells toward a pro-inflammatory state. This is characterized by increased IFNγ production upon target cell stimulation and elevated expression of inflammatory chemokines (*CCL3*, *CCL4*, *CCL5*) and *GZMA* at the transcriptomic level (Figures 4B, 5C). Importantly, this shift occurs without enhancement of cytotoxic function, as degranulation and target cell killing remain unchanged (Figures 5A,B). At the phenotypic level, T-BET overexpression promotes partial maturation, as evidenced by increased CD94 expression and strong induction of KIRs. However, it does not significantly increase CD16 or CD57 expression, indicating that terminal NK cell maturation is not achieved (Figure 4D).

Together, these findings support a model in which T-BET preferentially promotes inflammatory functional specialization, resulting in a functional dissociation from terminal cytotoxic NK cell maturation (Figure 6).

**FIGURE 6:**
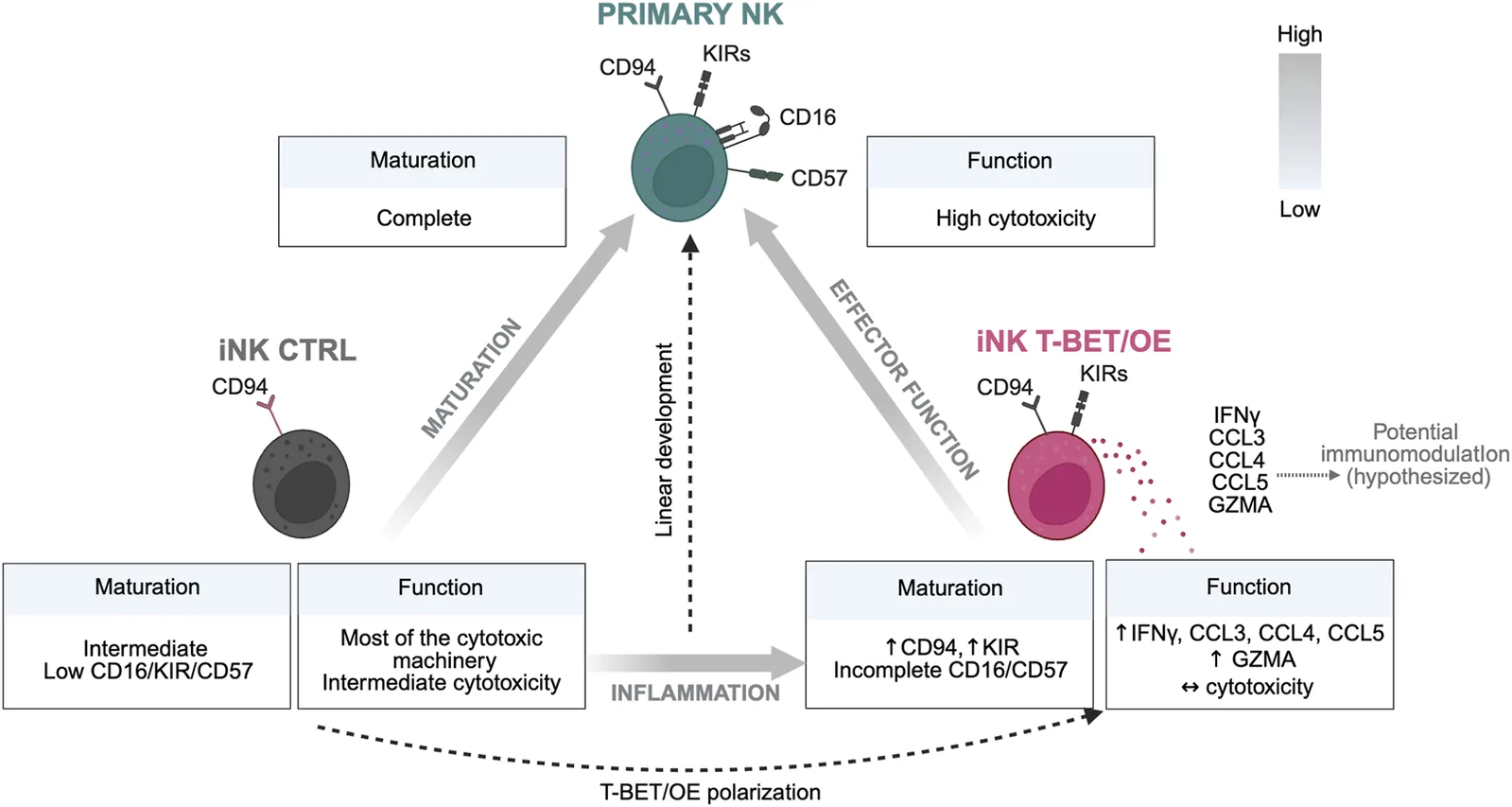
Conceptual model of T-BET-driven NK cell positioning along maturation and inflammatory axes. Schematic representation of NK cell differentiation and functional positioning. During NK cell development, immature NK cells are thought to progress through a linear fashion toward fully mature cytotoxic NK cells characterized by complete acquisition of CD16, KIR, and CD57 expression and high cytotoxic function. iNK CTRL align with an intermediate NK cell state, displaying partial cytotoxic machinery and incomplete acquisition of terminal maturation markers. T-BET overexpression redirects iNK cells toward a pro-inflammatory NK cell program, characterized by increased IFNγ production and elevated expression of inflammatory chemokines (CCL3, CCL4, CCL5) and GZMA, without enhancing cytotoxic function. This state is associated with partial phenotypic maturation, including increased CD94 and KIRs expression, but incomplete acquisition of CD16 and CD57. This model highlights a potential functional dissociation between inflammatory polarization and terminal NK cytotoxic maturation, suggesting possible immunomodulatory roles for T-BET-driven NK cells.

## 4 Discussion

iPSC-derived NK cells can be generated using a variety of differentiation protocols (Lupo et al., 2021; Zhu and Kaufman, 2019; Matsubara et al., 2019); however, their phenotypic, functional, and transcriptional characterization relative to primary NK cells remains incomplete. In this study, we show that feeder-free generated iNK cells are transcriptionally distinct from pNK cells and exhibit incomplete terminal maturation, with reduced expression of key maturation-associated markers despite acquisition of much of the cytotoxic machinery. While T-BET promoted aspects of phenotypic maturation, it was not sufficient to drive full terminal maturation. Instead, T-BET primarily induced a pro-inflammatory program, characterized by increased IFNγ production and chemokine expression, without enhancing cytotoxic activity. These findings indicate that T-BET can promote inflammatory polarization without driving full terminal NK-cell maturation or increasing cytotoxic function in human iPSC-derived NK cells.

Among the genes the most enriched in iNK cells, *LIN28B* was particularly notable. *LIN28B* is a well-established regulator of fetal hematopoiesis that is highly expressed in fetal but not adult hematopoietic tissues and functions as a molecular switch controlling developmental timing through the LIN28/let-7 axis. Ectopic expression of *LIN28B* has been shown to reprogram adult hematopoietic progenitors toward fetal-like lymphopoiesis, highlighting its role in maintaining developmental identity (Yuan et al., 2012). In addition to *LIN28B*, iPSC-derived NK cells displayed elevated expression of *IGF2BP1*, an oncofetal RNA-binding protein that is abundantly expressed during embryogenesis but largely silenced in adult tissues. Unlike transcription factors, *IGF2BP1* regulates post-transcriptional gene expression by stabilizing target mRNAs involved in developmental programs. *IGF2BP1* is highly expressed in fetal hematopoietic stem cells and functions downstream of the LIN28B/let-7 pathway, suggesting that the concurrent enrichment of *LIN28B* and *IGF2BP1* reflects persistence of an early developmental transcriptional program despite acquiring canonical NK-cell identity and most cytotoxic machinery (Ma et al., 2024).

T-BET is widely recognized as a key regulator of terminal NK cell maturation, with most evidence derived from murine models (Townsend et al., 2004; van Helden et al., 2015). In humans, NK cell maturation is characterized by the sequential acquisition of CD94, CD16, KIRs, and CD57, alongside a transition from CD56^bright^ to CD56^dim^ subsets (Yu et al., 2013; Béziat et al., 2010). T-BET expression increases along this maturation trajectory and is associated with the acquisition of KIRs and terminal differentiation features, often in coordination with EOMES (Collins et al., 2017). Consistent with this framework, we observed that T-BET overexpression in iPSC-derived NK cells enhanced CD94 and KIRs expression, supporting its role in promoting aspects of phenotypic maturation. However, T-BET was insufficient to drive full terminal differentiation, as evidenced by the lack of CD16 and CD57 acquisition. This suggests that while T-BET can induce components of the maturation program, it is not sufficient on its own to complete the transition to a fully mature NK cell state. These findings are partially consistent with previous reports showing that T-BET overexpression can promote CD94 and KIRs expression in human umbilical cord blood-derived NK cells (Kiekens et al., 2021). However, in contrast to this study, we did not observe a significant increase in CD16 expression, indicating that additional regulatory factors or environmental cues may be required to achieve full terminal maturation.

Cytotoxic function in NK cells relies on the expression of effector molecules such as granzyme B and perforin (Lieberman, 2003). T-BET has been implicated in the regulation of these genes and is thought to contribute to their expression during maturation, supporting a model in which cytotoxic function increases in parallel with phenotypic maturation (Townsend et al., 2004; Collins et al., 2017).In addition, NK cells are key producers of inflammatory cytokines, particularly IFNγ, and T-BET has been shown to play an important role in sustaining IFNγ production upon activation (Gordon et al., 2012; Townsend et al., 2004). These observations have led to the assumption that cytotoxic and inflammatory functions are coordinately regulated in NK cells. However, our data challenge this model. Here we showed that, while T-BET overexpression increased the expression of inflammatory mediators including IFNγ, it did not significantly increase the expression of granzyme B or perforin, nor did it enhance cytotoxic function under the conditions tested. Moreover, partial acquisition of maturation markers was not accompanied by increased cytotoxic machinery. Together, these findings indicate that T-BET can promote inflammatory transcriptional and functional features without a concomitant increase in cytotoxic function under the conditions tested, supporting a functional dissociation between inflammatory specialization and terminal cytotoxic maturation. Whether these processes are mechanistically independent remains to be established and would require independent experimental manipulation of the cytotoxic and inflammatory programs.

The fact that T-BET overexpression was not sufficient to induce full maturation in NK cells suggests that additional regulatory layers are required to complete this program. Terminal NK cell maturation is known to rely on the coordinated activity of multiple transcription factors, including EOMES (Collins et al., 2017; Knox et al., 2014), ZEB2 (van Helden et al., 2015), and B lymphocyte-induced maturation protein-1 (BLIMP1) (Smith et al., 2010), all of which are expressed at lower levels in iNK than in pNK clusters. In addition to transcriptional control, NK cell maturation is critically shaped by environmental cues. Cytokine signaling, particularly IL-15 trans-presentation (Lee et al., 2011), as well as interactions with stromal cells, play key roles in driving functional maturation. For instance, bone marrow stromal cells produce ligands such as growth arrest-specific 6 (Gas6) and Protein S (Pros1) that signal through TAM receptors (Tyro3, Axl, Mertk) and disruption of this pathway in mice leads to impaired expression of maturation-associated receptors, including natural killer group 2D (NKG2D) in NK cells (Caraux et al., 2006). In humans, Notch signaling has also been shown to contribute to NK cell maturation in a stage-dependent manner, promoting early development and later acquisition of CD16, KIRs expression, and functional competence (Felices et al., 2014). Such signals are likely to be not fully recapitulated *in vitro*, particularly in feeder-free differentiation systems, which may in turn explain the incomplete maturation observed in our model. The limited expression of CD16 and NKG2D in iNK cells further supports the idea that acquisition of these receptors may depend on specific microenvironmental interactions or additional soluble factors. Together, our data support a model in which NK cell maturation requires integration of transcriptional and environmental signals, in which T-BET preferentially drives inflammatory programs rather than terminal cytotoxic differentiation.

Beyond its established role in NK cell biology, T-BET acts across multiple innate and adaptive immune cell populations as a central regulator of type 1 immunity. In CD4^+^ T cells, T-BET drives helper T cell type 1 (TH1) differentiation by promoting IFNγ, CXCR3, CCL3, and CCL4 expression while repressing alternative TH2 and TH17 programs and stabilizing TH1 identity through chromatin remodeling at the *IFNG* locus (Sekimata et al., 2009). T-BET also promotes IFNγ production in γδ T cells and supports inflammatory activation of dendritic cells. In regulatory T cells (Treg), T-BET induces CXCR3-dependent trafficking to inflammation sites while in B cells it regulates IFNγ-dependent IgG2a class switching (Lazarevic et al., 2013; Huang and Bi, 2021). Interestingly, the consequences of T-BET activation appear highly context dependent across different immune lineages. In TH1 cells, dendritic cells, and NK cells, T-BET directly promotes inflammatory cytokine and chemokine programs, whereas in Treg and B cells it primarily facilitates adaptation to inflammatory environments. Consistent with this concept, our findings suggest that in iPSC-derived NK cells, T-BET preferentially drives an inflammatory transcriptional state rather than acting as a dominant regulator of terminal cytotoxic maturation. Together, these observations support a model in which T-BET functions broadly as an amplifier of inflammatory competence, while terminal maturation and cytotoxic differentiation remain dependent on additional lineage-specific regulatory networks.

Several limitations should be considered in our study. First, transcriptomic analyses were performed on a single iPSC-derived NK differentiation and one primary NK cell donor, which may limit generalization and does not fully capture inter-donor or inter-differentiation variability. Furthermore, because the scRNA-seq analysis was based on a single biological replicate per condition, the observed cell-level differences should be interpreted as descriptive rather than as statistically replicated population-level effects. Additional pNK donors expanded with IL-2 only (rather than IL-2 plus IL-15) showed consistent clustering in the supplementary UMAPs, and the transcriptomic observations were supported by consistent phenotypic and functional data across independent differentiations, strengthening the overall conclusions of the study. Second, our *in vitro* system does not fully recapitulate the complexity of NK cell development, particularly the contribution of microenvironmental cues present *in vivo*. As no model fully captures human NK ontogeny, caution is warranted when extrapolating these results. Third, we did not assess the signaling pathways underlying T-BET-mediated inflammatory programming. Functional analyses were focused on IFNγ, and the impact on other cytokines, including TNFα, remains to be explored. Moreover, the limited number of biological replicates and the variability observed in the chromium-release assay mean that small effects of T-BET overexpression on cytotoxicity cannot be completely excluded. Finally, although the T-BET/OE and CTRL populations were generated from the same parental iPSC14 line, thereby reducing variation attributable to differences in genetic background in the direct comparison, the use of a single iPSC line limits the generalizability of the T-BET phenotype across different iPSC lines. In addition, as the engineered populations (iNK CTRL and iNK T-BET/OE) were generated using transposon-mediated integration, differences in integration sites or copy numbers between populations cannot be fully excluded as potential sources of variability.

Interestingly, IFNγ transcript levels were not increased at steady state despite T-BET overexpression, although T-BET is known to directly activate IFNγ transcription (Jenner et al., 2009). Given that IFNγ production is also regulated post-transcriptionally (Anderson, 2008), our findings are consistent with a model in which T-BET may enhance cytokine production through mechanisms beyond transcriptional activation.

Finally, although T-BET induced robust KIRs expression, the functional implications remain unclear. KIRs contribute to NK cell education through interactions with MHC class I (Anfossi et al., 2006). Unfortunately, there is no standard *in vitro* model for NK cell education, limiting conclusions regarding their contribution to functional NK cell competence. Furthermore, assessing whether the CD94^+^KIR^+^ subset displays enhanced functionality would require functional analyses specifically restricted to relatively small cell populations, making robust functional characterization technically challenging.

Future studies should further investigate the mechanisms through which T-BET selectively promotes inflammatory programming in iPSC-derived NK cells. It will be important to determine whether T-BET directly regulates chromatin accessibility and transcription of inflammatory genes or instead potentiates cytokine-responsive pathways such as IL-12/STAT4 or IFNγ/STAT1 signaling. Given the central role of IL-15 and IL-2 during NK cell differentiation (Steel et al., 2012), it will also be relevant to assess whether T-BET modifies responsiveness to these cytokines through regulation of cytokine receptor expression or downstream signaling pathways. In parallel, additional studies are needed to determine how this T-BET-driven inflammatory NK cell state influences surrounding immune populations. Co-culture systems with dendritic cells, macrophages, or T cells could help define whether these cells possess enhanced immunomodulatory properties capable of promoting antigen presentation, T-cell activation, or broader anti-tumor immune responses.

From a translational perspective, our findings suggest that T-BET-driven iPSC-derived NK cells represent a functionally specialized inflammatory NK-cell state rather than a purely cytotoxic NK-cell product. Although T-BET overexpression did not enhance direct killing capacity, it strongly increased IFNγ and inflammatory chemokine production, which may promote immune-cell recruitment, antigen presentation, and T-cell activation within the tumor microenvironment. These properties could be particularly relevant to combined cell immunotherapy settings, where inflammatory NK cells may enhance responsiveness to immune checkpoint blockade or support T cell-based therapies by promoting local immune activation. More broadly, our results support the concept that NK-cell engineering strategies can be tailored toward distinct therapeutic objectives, including direct cytotoxicity or inflammatory immune modulation depending on the clinical context.

## 5 Conclusion

In summary, our study demonstrates that T-BET selectively promotes an inflammatory effector program in iPSC-derived NK cells without inducing full terminal cytotoxic maturation and suggests that engineered iPSC-derived NK cells may be directed toward distinct functional states rather than a single mature endpoint. Together, our results provide new insights into the transcriptional regulation of human NK-cell maturation and functional specialization, supporting the development of tailored NK-cell engineering strategies for immunotherapy.

## Data Availability

The original contributions presented in the study are publicly available in the NCBI repository (GEO accession GSE351776).
