# Supplementary material for: T-BET promotes inflammatory specialization without driving terminal maturation of iPSC-derived NK cells
Source: Front Pharmacol. 2026 Oct 27;17:1952429. doi: 10.3389/fphar.2026.1952429 (PMC13749396; doi:10.3389/fphar.2026.1952429)
Supplement: Supplementary file 1 [file Supplementaryfile1.docx]

Supplementary Material

# 1 SUPPLEMENTARY DATA

**Supplementary Figure S1. Integration of iPSC-derived NK cells with additional peripheral NK-cell donors and identification of cluster-specific transcriptional signatures.**

(A) Integrated UMAP of iPSC-derived NK (iNK) cells and three peripheral NK (pNK) donors without batch correction, colored by sample identity. iNK cells and pNK donor 1 were cultured with IL-2 and IL-15, whereas pNK donors 2 and 3 were cultured with IL-2 only before scRNA-seq analysis. (B) The same UMAP colored according to unsupervised clustering. (C) Split UMAP showing the distribution of each sample across the identified clusters. (D) Heatmap showing the top differentially expressed genes between iNK cells and the matched pNK donor (donor 1), both cultured under identical conditions (IL-2 + IL-15), corresponding to the dataset used for the analyses presented in Fig. 1. (E) Heatmap showing the top marker genes defining each transcriptional cluster from the matched iNK versus pNK donor 1 dataset used in Fig. 1.

**Supplementary Figure S2. Additional characterization of iPSC-derived NK cells and validation of inducible constructs.**

(C) Schematic map of the PiggyBac transposase construct used for genomic integration . (B-D) Schematic representation of the inducible constructs used in this study, including the rtTA cassette (B), CTRL construct (TRE_mCherry) (C), and T-BET/OE construct (TRE_T-BET_T2A_mCherry) (D). (E) Representative flow cytometry plots from independent replicates showing doxycycline-inducible T-BET and mCherry expression in CTRL and T-BET/OE iPSCs cultured in the presence (+DOX) or absence (−DOX) of doxycycline.

**Supplementary Figure S3. Additional phenotypic characterization of T-BET-overexpressing iPSC-derived NK cells.**

(A) Percentage of NKG2A⁺ cells across differentiation stages in iNK CTRL, iNK T-BET/OE, and pNK cells. (B) Percentage of Nkp80⁺ cells across differentiation stages in iNK CTRL, iNK T-BET/OE, and pNK cells. (C) Total number of suspension cells generated from four 6-well plates during independent iNK CTRL and iNK T-BET/OE differentiations across four batches. Live cell counts were taken at the end of NK lineage commitment before replating the cells for late-stage NK cell differentiation. (D) Representative histograms from independent differentiation replicates showing expression of CD94, CD16, and KIR2DL2/3 in iNK CTRL, iNK T-BET/OE, and pNK cells at late NK stage. Data in (A-B) are shown as individual values with mean ± SD. iNK CTRL, N=3 independent differentiations; iNK T-BET/OE, N=3 independent differentiations; pNK,N=7 healthy donors. Data were analyzed using two-way repeated measures ANOVA with Šídák’s multiple comparisons test for iNK CTRL vs T-BET/OE. At the late NK stage, comparisons including pNK were performed using a Kruskal-Wallis test with Dunn’s multiple comparisons.

**Supplementary Figure S4. Additional transcriptomic and phenotypic characterization of iNK CTRL, iNK T-BET/OE, and primary NK cells.**

(A) UMAP visualization of scRNA-seq data showing the distribution of iNK T-BET/OE and pNK donor 1 (D1) cells (left), iNK CTRL and pNK donor 1 (D1) cells (middle), and an integrated UMAP including iNK CTRL, iNK T-BET/OE, and pNK cells from three independent healthy donors (D1–D3)(right).

(B) Violin plots showing normalized expression levels of *IFNG*, *XCL1*, and *NKG7* across iNK CTRL, iNK T-BET/OE, and pNK cells. (C) Heatmap showing the percentage of cells expressing the indicated markers in iNK CTRL, iNK T-BET/OE, and pNK populations.

For scRNA-seq analyses (A–B), data are derived from one representative differentiation for iNK CTRL and iNK T-BET/OE (N = 1 each) and three independent healthy pNK donors (D1–D3). iNK cells (iNK CTRL and iNK T-BET/OE) and pNK donor 1 were cultured with IL-2 and IL-15, whereas pNK donors 2 and 3 were cultured with IL-2 only before scRNA-seq analysis. For phenotypic analysis (C), data represent mean values from iNK CTRL (N = 3 independent differentiations), iNK T-BET/OE (N = 3 independent differentiations), and pNK (N = 7 healthy donors).

**Supplementary Figure S5. Flow cytometry gating strategy for inducible system validation in iPSCs.**

(A) Gating strategy used for analysis of EBFP2 and mCherry expression in CTRL and T-BET/OE iPSCs cultured in the presence (+DOX) or absence (−DOX) of doxycycline. Live single cells were selected based on FSC/SSC properties, singlet discrimination, and live/dead exclusion. EBFP2 gating was established using WT iPSCs as negative controls. mCherry gating was determined using fluorescence minus one (FMO) controls. Representative contour plots are shown.

(B) Gating strategy used for intracellular T-BET staining in CTRL and T-BET/OE iPSCs cultured in the presence (+DOX) or absence (−DOX) of doxycycline. Live single cells were selected as described in (A). T-BET positivity was defined using WT iPSCs as negative controls following intranuclear staining. Representative contour plots are shown.

**Supplementary Figure S6. Flow cytometry gating strategy for phenotypic and functional analyses of iNK cells.**

(A) Gating strategy used for phenotypic analysis of iNK cells. Live single cells were selected based on FSC/SSC properties, singlet discrimination, and live/dead exclusion. CD45⁺CD56⁺ NK cells were gated using controls stained only with live/dead. Subsequently, mCherry⁺ cells were gated using WT iNK cells as a negative control. Finally, phenotypic marker expression (example shown for CD94) was analyzed within the CD45⁺CD56⁺mCherry⁺ population using an iNK sample stained only for CD45, CD56 and CD3 as a negative control. Representative contour plots are shown.

(B) Gating strategy used for degranulation and intracellular IFNγ production assays. Live single cells were selected as described in (A), followed by gating on CD45⁺CD56⁺CD3⁻ NK cells. mCherry⁺ cells were selected for analysis of iNK CTRL and iNK T-BET/OE cells, while primary NK (pNK) cells served as negative controls for mCherry expression. CD107a and IFNγ positivity were determined using fluorescence minus one (FMO) controls. Representative contour plots are shown.

**Supplementary Figure S7. Quality control and filtering of single-cell RNA sequencing datasets.**

(A) Violin plots showing quality control metrics before and after filtering for iNK (WT), iNK CTRL, iNK T-BET/OE, and pNK samples. Metrics include the number of detected genes per cell (nFeature_RNA), total RNA counts per cell (nCount_RNA), and percentage of mitochondrial transcripts (percent.mt). Cells with low transcript counts, low gene numbers, or high mitochondrial transcript content were excluded prior to downstream analysis. (B) Summary table showing the number of cells retained before and after quality filtering for each sample.

**Supplementary Figure S8. Characterization of pluripotency and trilineage differentiation potential of engineered iPSC lines.**

(A) Flow cytometry analysis of pluripotency marker expression in CTRL and T-BET/OE iPSC lines. (B) Trilineage differentiation capacity of CTRL, T-BET/OE and WT iPSC lines assessed by flow cytometry following directed differentiation toward mesoderm, endoderm, and ectoderm lineages. Mesoderm differentiation was evaluated by expression of CD56 and Brachyury, endoderm differentiation by FOXA2 and SOX17 expression, and ectoderm differentiation by PAX6 and NESTIN expression. Data are shown as mean ± SEM of experimental triplicates (n=3) from one representative experiment (N=1).

**Supplementary Figure S9. Genetic characterization of iPSC lines used for NK cell differentiation.**

(A) KIR genotyping of iPSC lines iPSC9, iPSC7, and iPSC14. Presence (POS) or absence (NEG) of inhibitory and activating KIR genes was determined for each donor-derived iPSC line. (B) High-resolution HLA typing of the iPSC14 line showing HLA class I and class II alleles. (C) Predicted KIR-HLA interactions in iPSC14 based on KIR genotype and HLA ligand expression. Predicted inhibitory and activating interactions are indicated according to known KIR ligand specificities. (D) Summary of karyotyping analysis for iPSC lines used in this study. No chromosomal abnormalities were detected in any of the analyzed lines. (E) Representative KaryoStat profiles of iPSC9, iPSC7, and iPSC14 confirming genomic stability of the iPSC lines prior to differentiation.

# 2 SUPPLEMENTARY EXPERIMENTAL PROCEDURES

**Supplementary Table S1. List of antibodies used in the study**

| **Target** | **Supplier** | **Cat** | **Clone** | **Fluorochrome** | **Dilution** |
| --- | --- | --- | --- | --- | --- |
| CD56 | BioLegend | 318332 | HD56 | APC-Cy7 | 1:20 |
| CD45 | BioLegend | 304024 | HI30 | AF700 | 1:50 |
| CD94 | BioLegend | 305512 | DX22 | AF647 | 1:40 |
| CD314  (NKG2D) | BD Biosciences | 562365 | 1D11 | PE-Cy7 | 1:50 |
| CD3 | BioLegend | 300310 | HIT3a | PE-Cy5 | 1:100 |
| CD226  (DNAM) | BD Biosciences | 559789 | DX11 | PE | 1:10 |
| CD57 | BioLegend | 393303 | QA17A04 | BV605 | 1:320 |
| CD159  (NKG2C) | BD Biosciences | 749685 | 134591 | BUV737 | 1:20 |
| CD159a  (NKG2A) | BD Biosciences | 752302 | 131411 | BUV615 | 1:50 |
| CD2 | BD Biosciences | 750642 | S5.2 | BUV496 | 1:40 |
| CD16 | BD Biosciences | 563784 | 3G8 | BUV395 | 1:50 |
| NKp80 | BioLegend | 346708 | 5D12 | APC | 1:20 |
| CD335  (NKp46) | BD Biosciences | 562101 | 9E2 | PE-Cy7 | 1:20 |
| CD253  (TRAIL) | BD Biosciences | 559789 | DX11 | PE | 1:20 |
| CD11a  (LFA-1alpha) | BD Biosciences | 742626 | HI111 | BV786 | 1:40 |
| CD336  (NKp44) | BD Biosciences | 744303 | p44-8 | BV711 | 1:150 |
| CD337  (NKp30) | BD Biosciences | 563384 | P30-15 | BV605 | 1:50 |
| CD178  (FasL) | BD Biosciences | 748972 | NOK-1 | BUV737 | 1:20 |
| CD244  (2B4) | BioLegend | 329516 | C1.7 | PerCP-Cy5.5 | 1:160 |
| CD158ah  (KIR2DL1/S1) | BC Immunotech | A66899 | / | PE-Cy7 | 1:20 |
| CD158e1e2  (KIR3DL1/S1) | BC Immunotech | IM3292 | / | PE | 1:20 |
| CD158b  (KIR2DL2/3) | BD Biosciences | 743454 | CH-L | BV711 | 1:20 |
| T-BET | BD Biosciences | 561316 | O4-46 | PerCP-Cy5.5 | 1:50 |
| SOX2 | BD Biosciences | 562139 | O30-678 | AF647 | 1:50 |
| SSEA-4 | BD Biosciences | 560126 | MC813-70 | FITC | 1:50 |
| OCT4 | BioLegend | 653704 | 3A2A20 | PE | 1:50 |
| TRA-1-60 | BD Biosciences | 563878 | TRA-1-60 | BUV395 | 1:50 |
| SOX17 | R&D Technologies | IC1924A | / | APC | 1:50 |
| FoxA2 | BD Biosciences | 561589 | N17-280 | PE | 1:50 |
| Brachyury | R&D Technologies | IC2085A | Polyclonal | APC | 1:50 |
| CD56 | BioLegend | 318306 | HD56 | PE | 1:50 |
| Nestin | BD Biosciences | 561231 | 25 | PerCP-Cy5.5 | 1:50 |
| Pax6 | BD Biosciences | 561552 | O18-1330 | PE | 1:50 |
| IFNγ | BioLegend | 506510 | B27 | APC | 1:400 |
| CD107a | BioLegend | 328618 | H4A3 | PE-Cy7 | 1:800 |

## 2.1 Plasmids

The CAG_HyperPB plasmid shown in Supplementary Figure S1C was kindly provided by Jere Weltner (University of Helsinki). To generate the CAG_rtTA_T2A_EBFP2 construct (Supplementary Figure S1D), a synthetic insert flanked by NheI and XhoI restriction sites and containing the genes of interest was synthesized by GenScript. The insert was subsequently cloned into the PiggyBac backbone plasmid PB-CA-rtTA Adv (gift from Andras Nagy; Addgene plasmid #20910; RRID:Addgene_20910). Similarly, the TRE_mCherry_EFS_PuroR and TRE_T-BET_mCherry_EFS_PuroR plasmids (Supplementary Figure S1D) were generated using a multistep cloning strategy. First, a synthetic TRE_MCS1_T2A_MC2 cassette flanked by NheI/XhoI restriction sites was synthesized by GenScript and inserted into the PB-CA-rtTA Adv backbone. The TRE_mCherry_EFS_PuroR construct was then generated by cloning a synthetic mCherry sequence into the intermediate plasmid using AgeI/XbaI restriction sites. For the TRE_T-BET_mCherry_EFS_PuroR construct, a condon optimized T-BET sequence was inserted using AgeI/XbaI restriction sites, followed by insertion of mCherry using BamHI/EcoRI restriction sites.

## 2.2 Mutagenic detection of iPSC

Genomic stability of iPSC lines was assessed using the KaryoStat+ assay (Thermo Fisher Scientific), performed by an external service provider. Briefly, genomic DNA was isolated from iPSCs and subjected to microarray-based analysis enabling genome-wide assessment of copy number variations and chromosomal aberrations. The assay provides detection of aneuploidies and submicroscopic genomic alterations across the genome with a resolution of approximately 1-5 Mb.

## 2.3 KIR and HLA typing

Genomic DNA was extracted from iPSCs using the AllPrep DNA/RNA/Protein Mini Kit (Qiagen, CAT#80004) according to the manufacturer’s instructions. DNA quality and concentration were assessed prior to downstream applications using a nanodrop-based quantification. KIR genotyping was performed by an external provider using PCR-SSP–based methods (Olerup SSP® KIR Genotyping, CareDx) according to standard protocols . HLA typing was conducted by the same provider using validated clinical genotyping methods.

## 2.4 Trilineage differentiation

Pluripotency of iPSCs was assessed using a trilineage differentiation assay (STEMdiff™ Trilineage Differentiation Kit; STEMCELL Technologies, Cat# 05230) according to the manufacturer’s instructions. Briefly, iPSCs were induced toward ectoderm, mesoderm, and endoderm lineages under defined culture conditions, and lineage specification was evaluated via flow cytometry analysis using established markers.
